# Simulation and surface topology of activity of pyrazoloquinoline derivatives as corrosion inhibitor on the copper surfaces

**DOI:** 10.1038/s41598-021-91159-6

**Published:** 2021-06-09

**Authors:** Razieh Razavi, Savaş Kaya, Mahboobeh Zahedifar, Sayed Ali Ahmadi

**Affiliations:** 1 Department of Chemistry, Faculty of Science, University of Jiroft, Jiroft, Iran; 2 Department of Pharmacy, Health Services Vocational School, Sivas Cumhuriyet University, 58140 Sivas, Turkey; 3 Department of Chemistry, Kerman Branch, Islamic Azad University, Kerman, Iran

**Keywords:** Chemistry, Engineering

## Abstract

In the present study, corrosion inhibition performances of some pyrazolo [3,4-b] quinoline-3,5-dione derivatives against the corrosion of copper metal were investigated using B3LYP/6-311++g(d,p) calculation level in aqueous media. Additionally, interaction energies were calculated for all the pyrazoloquinoline derivatives compounds. In the calculations it is observed that studied molecules adsorb on metal surface with the help of electron donor heteroatoms in their molecular structures. Chemical thermodynamic parameters regarding the interaction between inhibitor molecule and copper surface were estimated and discussed. Density of the electron profile analysis and chemical electrostatic potential of nuclear charges in the molecule were applied to consider the nature of a number of probable interactions between Cu metal surface and inhibitors in terms of bond critical point (BCP). Calculated quantum chemical parameters showed that the pyrazoloquinoline derivatives including the OH and NO_2_ exhibit high inhibition performance.

## Introduction

The heterocyclic compounds containing pyrazole and quinoline systems, such as pyrazolo[1,5-] pyrimidine, pyrazolopyridine, pyrazoloquinolone, pyrazolo[4,3-c]quinoline and pyrazolo[1,5] quinazoline are widely considered in the drug design and medicine. Pyrazolo[3,4- b]quinoline system exhibits antiviral, antimalarial, and anti-inflammatory activity^1^ and high corrosion inhibition activity^2^. It should be noted that in consideration π bonds and heteroatoms in the molecular structure of quinoline and pyrazole, corrosion inhibition effects of these molecules against the corrosion of various metals^3–10^ have been investigated previously^11,12^. Corrosion inhibition effect strongly depends on adsorption of the inhibitor molecules on the surface of metal. It has been shown that corrosion inhibition is influenced by different factors like type of corrosive material, chemical essence of the metal and structure of the chemical component applied as active inhibitor. These parameters are directly associated with adsorption capability and the nature of the interaction between metal and inhibitor^13–15^.

Through molecular simulation and quantum chemical techniques, corrosion inhibition performances of molecules can be linked to chemical and electronic molecular properties^12–32^. In this study, corrosion inhibition activity of pyrazolo[3,4-b] quinoline-3,5-dione derivatives (R = Ph, 2-(NO_2_)C_6_H_4_, 4-(N, N-di-Me)C_6_H_4_, 2,4-di-ClC_6_H_3_, 2-(OH)C_6_H_4_, 4-(OH)C_6_H_4_, 4-(Me)C_6_H_4_, 4-(OCH_3_)C_6_H_4_, 4-(Cl)C_6_H_4_) on Cu surface was investigated. The power of the interactions between the mentioned molecules and said metal surface were highlighted and presented. Charge densities were calculated and discussed. The interface was created for simulation of molecule/metal surface. Reactivities of of Cu surface with the molecule were also estimated. It is well-known that sometimes, experimental techniques used in corrosion inhibition studies can be expensive and time-consuming. For that reason, theoretical approaches started to be preferred frequently in the analysis of corrosion inhibition performances of organic and inorganic molecules. The novelty of this study is that it is first theoretical attempt analyzing the corrosion inhibition performances of selected inhibitor molecules against the corrosion of copper metal. Therefore, theoretical data and discussions presented about some pyrazolo [3,4-b] quinoline-3,5-dione derivatives the in this study will shed light to the experimental and theoretical corrosion studies in the future.

## Results

**Figure 1:**
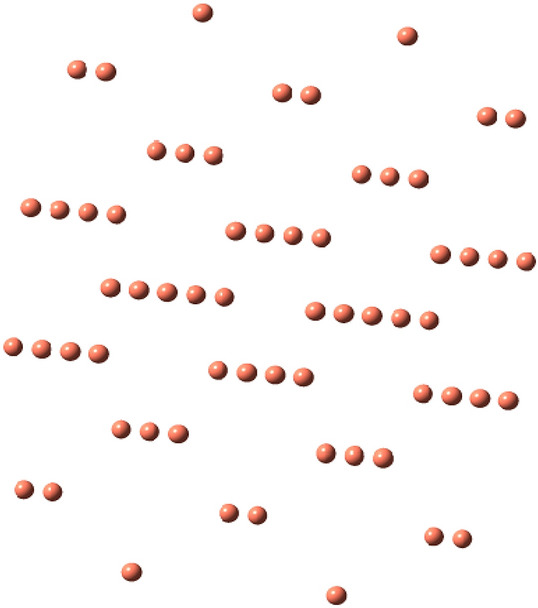
Optimized Cu (fcc) structure (https://chemistry.com.pk/software/free-download-chemdraw-ultra-12/, https://gaussview.software.informer.com/5.0/).

**Figure 2:**
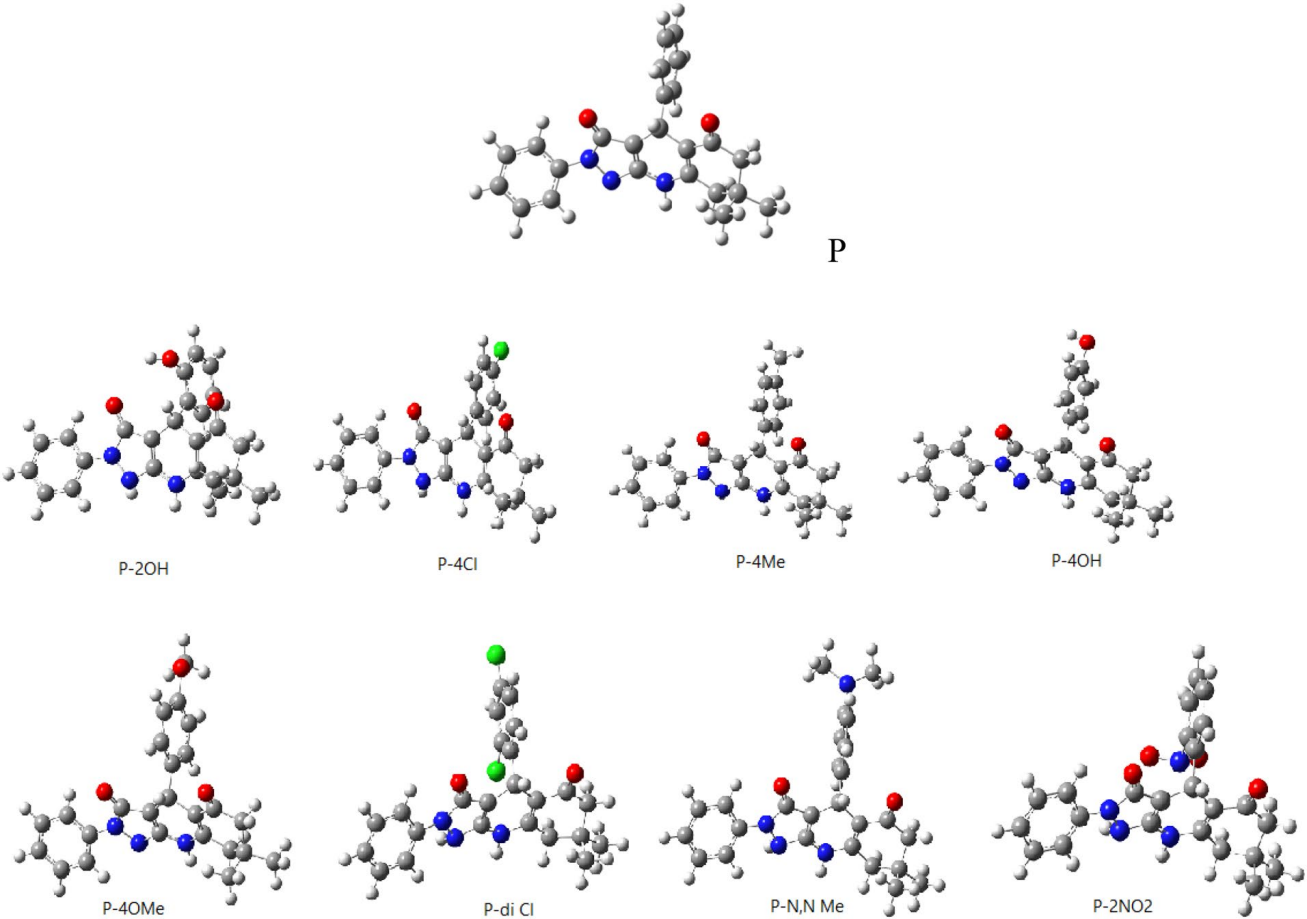
Optimized pyrazoloquinolines derivatives’ structure (R = Ph, 2-(NO_2_)C_6_H_4_, 4-(N,N-di-Me)C_6_H_4_, 2,4-di-ClC_6_H_3_, 2-(OH)C_6_H_4_, 4-(OH)C_6_H_4_, 4-(Me)C_6_H_4_, 4-(OCH_3_)C_6_H_4_, 4-(Cl)C_6_H_4_)(https://chemistry.com.pk/software/free-download-chemdraw-ultra-12/, https://gaussview.software.informer.com/5.0/).

One of the important goals of this study is to illustrate electronic interactions between Cu(fcc) surface with of pyrazoloquinoline derivatives (R = Ph, 2-(NO_2_)C_6_H_4_, 4-(N,N-di-Me)C_6_H_4_, 2,4-di-ClC_6_H_3_, 2-(OH)C_6_H_4_, 4-(OH)C_6_H_4_, 4-(Me)C_6_H_4_, 4-(OCH_3_)C_6_H_4_, 4-(Cl)C_6_H_4_) as potential corrosion inhibitors. Electrochemical and thermodynamic principles are the original principles of corrosion behavior defining transformation of the alloys and metals into stable states. Figures 1 and 2 show all the optimized structures. Nitrogen and oxygen heteroatoms in pyrazoloquinoline molecule could interact with the Cu surface. Also, electrons of benzene ring on R derivatives interacted with surface atom of the metal compound in parallel and perpendicular orientations. All the parallel and perpendiculars orientations were simulated for chemical inhibitor molecules as exemplified in Fig. 4. Table 1 shows the exchanges of Gibbs free energy of Cu complexes with the chemical inhibitor pyrazoloquinoline molecule derivatives. Optimized sides of the inhibitor molecules are perpendicular to interact with Cu surface. Pyrazole ring has an important role in terms of the interaction in Cu complexes.

**Table 1:** Gibbs free energy of Cu and of pyrazoloquinolines derivatives’ structure (R = Ph, 2-(NO2)C6H4, 4-(N,N-di-Me)C6H4, 2,4-di-ClC6H3, 2-(OH)C6H4, 4-(OH)C6H4, 4-(Me)C6H4, 4-(OCH3)C6H4, 4-(Cl)C6H4) and Cu complexes.

Cu/R Complexes	Ph	2-(NO2)C6H4	4-(N,N-di-Me)C6H4	2,4-di-ClC6H3	2-(OH)C6H4	4-(OH)C6H4	4-(Me)C6H4	4-(OCH3)C6H4	4-(Cl)C6H4
∆*G*/Kcal/mol	− 4.87	− 5.17	− 4.82	− 4.72	− 5.35	− 5.06	− 4.41	− 4.96	− 4.76

**Figure 3:**
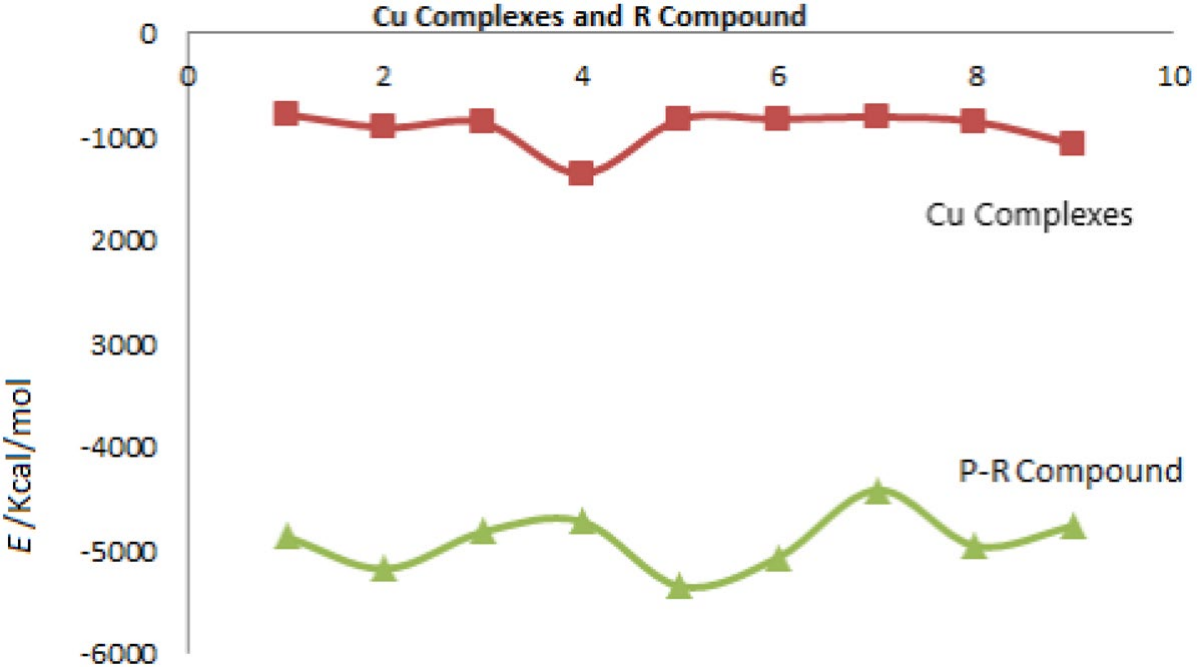
Difference between energy level of pyrazoloquinolines derivatives’ structure (R = Ph, 2-(NO_2_)C_6_H_4_, 4-(N,N-di-Me)C_6_H_4_, 2,4-di-ClC_6_H_3_, 2-(OH)C_6_H_4_, 4-(OH)C_6_H_4_, 4-(Me)C_6_H_4_, 4-(OCH_3_)C_6_H_4_, 4-(Cl)C_6_H_4_) and Cu complexes (Microsoft Excel 2010).

Via reaction thermodynamics, direction of the reaction can be predicted and they can be applied to investigate and estimate whether corrosion behavior on metal surfaces is probable or not theoretically. According to the results presented in Table 1, all the Cu complexes have negative Gibbs free energy showing that all the derivatives can be effective against the corrosion. Figure 3 and Table 2 illustrate different ranges of energy in pyrazoloquinoline derivatives and Cu complexes of pyrazoloquinoline derivatives. The derivatives containing –NO_2_ and –OH functional groups have the minimum negative value of Gibbs free energy and they have a strong interaction with the surface compared to other pyrazoloquinoline derivatives. Figure 3 shows the pyrazoloquinoline derivative inhibitors with electron-rich groups. Spatial congestion may cause –NO2 and –OH to have minimum Gibbs free energy leading to the best stability in water phase.

**Table 2:** Gibbs free energy.

_R/Compunds_	_∆*G* (cal/mol)_	_HOMO (a.u.)_	_LUMO (a.u.)_
Ph	− 780.37	− 0.22198	− 0.06357
2-(NO_2_)C_6_H_4_	− 908.2	− 0.2136	− 0.155798
4-(N,N-di-Me)C_6_H_4_	− 863.99	− 0.16813	− 0.05483
2,4-di-ClC_6_H_3_	− 1356.75	− 0.21018	− 0.05595
2-(OH)C_6_H_4_	− 827.14	− 0.21086	− 0.05429
4-(OH)C_6_H_4_	− 827.14	− 0.21137	− 0.05684
4-(Me)C_6_H_4_	− 804.62	− 0.20843	− 0.05402
4-(OCH_3_)C_6_H_4_	− 851.79	− 0.2117	− 0.05726
4-(Cl)C_6_H_4_	− 1068.32	− 0.20883	− 0.05656

## Discussion

Essentially, corrosion consists of two half-cell electrochemical reactions. Anodic and cathodic reactions involve leaving and taking up of the unrestricted and free electron by ionization of the metal or alloys nevertheless, cathodic reaction implicates taking up of free electrons by the dissolved oxygen in electrolyte molecules. On the oxidation reaction and solution reaction, free electrons are shaped although reduction reaction occurs later in which the electrons have been accepted. Corrosion behavior of metals and alloys can be simply tested with the using of the electrochemical techniques^33^. Because of adsorption of chemical inhibitor in solution/metal interface, corrosion of the alloys and metals can be prevented. Inhibition effect of the inhibitor molecule occurs at interface that includes electron transfer and adsorption of corrosion inhibitors on the metal surface. It is well-known that Density Functional Theory considers the electron density (*ρ*)^34^ to predict the chemical reactivity and stability of molecules. To predict the electron donating and electron accepting capabilities of pyrazolo[3,4-b] quinoline-3,5-dione derivatives (R = Ph, 2-(NO_2_)C_6_H_4_, 4-(N,N-di-Me)C_6_H_4_, 2,4-di-ClC_6_H_3_, 2-(OH)C_6_H_4_, 4-(OH)C_6_H_4_, 4-(Me)C_6_H_4_, 4-(OCH3)C_6_H_4_, 4-(Cl)C_6_H_4_), HOMO and LUMO energies of the mentioned species were calculated as shown in Table 2.

In the light of Koopmans Theorem, ionization energy (I) and electron affinities (A) can be roughly predicted from the energies of HOMO and LUMO orbitals regarding to the studied molecules using the relations $I = - E_{HOMO}$ and $A = - E_{LUMO}$. The following equations are used in the calculation of the quantum chemical parameters like chemical potential (µ), electronegativity (χ) and chemical hardness (η).

1 $$ \mu = - \chi = - \left( {\frac{I + A}{2}} \right), $$

2 $$ \eta = \frac{I - A}{2}. $$

Gazquez et al.^35^ proposed two new parameters called as electrodonating power (ω^−^) and electroaccepting power (ω^+^) to predict the electron donating and electron accepting abilities of chemical species. The mentioned parameters based on ground state ionization energy (I) and ground state electron affinity (A) of compounds are calculated via the following formulae.

3 $$ \omega^{ + } = (I + 3A)^{2} /(16(I - A)), $$

4 $$ \omega^{ - } = (3I + A)^{2} /(16(I - A)). $$

Some electronic structure principle known Maximum Hardness Principle^36^, Minimum Polarizability Principle^37^ and Minimum Electrophilicity Principle^38^ provide useful explanations in terms of the comparison of stabilities or reactivities of molecules. Chemical hardness^39^ is reported the reluctance against the polarization of electron cloud of chemical species. According to Maximum Hardness Principle formulated by Pearson based on chemical hardness concept, “there seems to be a rule of nature that molecules arrange themselves so as to be as hard as possible”. This means that hardness can be considered as an indicator of the stability. It is well-known that there is an inverse relation between hardness and polarizability because hard molecules have high polarizability values. Minimum Polarizability Principle states that in a stable state, polarizability is minimized. Minimum Electrophilicity Principle is another electronic structure principle taken into consideration for chemical reactivity analysis. According to this principle “the sum of the electrophilicity indices, of the reaction products will be smaller than that of the reactants.” In a recent paper, Szentpaly et al.^40^ reinvestigated the Minimum Electrophilicity Principle and presented new theorems and guiding rules regarding to its validity and limitations. In the same paper, authors proposed Maximum Composite Hardness Rule depending on Kaya’s composite descriptor, namely ${\upeta }_{{\text{M}}} {\text{/V}}_{{\text{m}}}^{{1/3}}$ ratio. It is apparent from the data presented in Table 3, pyrazoloquinolines derivatives including –OH, –OCH_3_ and –NO_2_ groups will be more effective against the corrosion of metal surfaces. For instance, the most polarizable molecules are 4-(OCH_3_)C_6_H_4_ and 2-(OH)C_6_H_4_. If so, these molecules are effective corrosion inhibitors. On the other hand, electrodonating abilities calculated Gazquez’s equations are quite high. It should be noted that Maximum Hardness Principle and Maximum Composite Hardness Rule support that 2-(NO_2_)C_6_H_4_ molecule also will be effective against the corrosion of metal surfaces.

**Table 3:** Quantum descriptor of pyrazoloquinolines derivatives.

R-compounds	*IP* (eV)	*EA* (eV)	*χ* (eV)	*η* (eV)	*µ* (eV)	α	ω^+^	ω^−^	V_m_ (nm^3^)	η/V_m_^1/3^	Dipole moment (Debye)
Ph	3.6	1.04	2.32	1.28	− 2.32	0.347	1.10	3.42	0.8113	1.372	8.44
2-(NO_2_)C_6_H_4_	3.5	2.5	3	0.5	− 3	0.239	7.56	10.56	0.9060	0.517	5.79
4-(N,N-di-Me)C_6_H_4_	2.7	0.89	1.79	0.9	− 1.79	0.367	1.00	2.79	0.9019	0.932	8.92
2,4-di-ClC_6_H_3_	3.44	0.91	2.17	1.26	− 2.17	0.327	0.94	3.12	0.9562	1.279	7.94
2-(OH)C_6_H_4_	3.45	0.88	2.16	1.28	− 2.16	0.396	0.90	3.07	0.8450	1.354	9.62
4-(OH)C_6_H_4_	3.46	0.93	2.19	1.26	− 2.19	0.389	0.96	3.16	0.8450	1.333	9.47
4-(Me)C_6_H_4_	3.41	0.885	2.14	1.262	− 2.14	0.366	0.91	3.06	0.8408	1.337	8.91
4-(OCH_3_)C_6_H_4_	3.47	0.93	2.2	1.27	− 2.2	0.438	0.96	3.16	0.8745	1.328	10.66
4-(Cl)C_6_H_4_	3.42	0.92	2.17	1.25	− 2.17	0.390	0.95	3.12	0.8838	1.303	9.48

DFT is an easy technique to obtain useful information about molecular structure and activities of corrosion inhibitors^10,41,42^ against the corrosion of metal surfaces. Simulation techniques have been developed as a powerful tool to understand the nature of the interactions between inhibitor molecules and metal surfaces^43^. Set orbit of linear combinations of geosynchronous functions was used for the basis aimed at electronically computing the design. Linear combination of several codgers was used due to accurate representation of atomic orbitals.

HOMO and LUMO energy levels are considered as important tools in the chemical reactivity analysis in Molecular Orbital Theory. Fukui^44^ acknowledged that frontier orbitals play important roles in the chemical reactions. Corrosion inhibition performances of molecules can be easily predicted within the framework of the energy levels of HOMO and LUMO orbitals. It should be noted that the molecules having high HOMO energy values can be easily adsorbed on metal surfaces because they give the electrons easily to metals. Namely, E_HOMO_ represents the tendency to donate the electrons while E_LUMO_ is a measure of electron accepting capability of corrosion inhibitors^45^.

**Figure 4:**
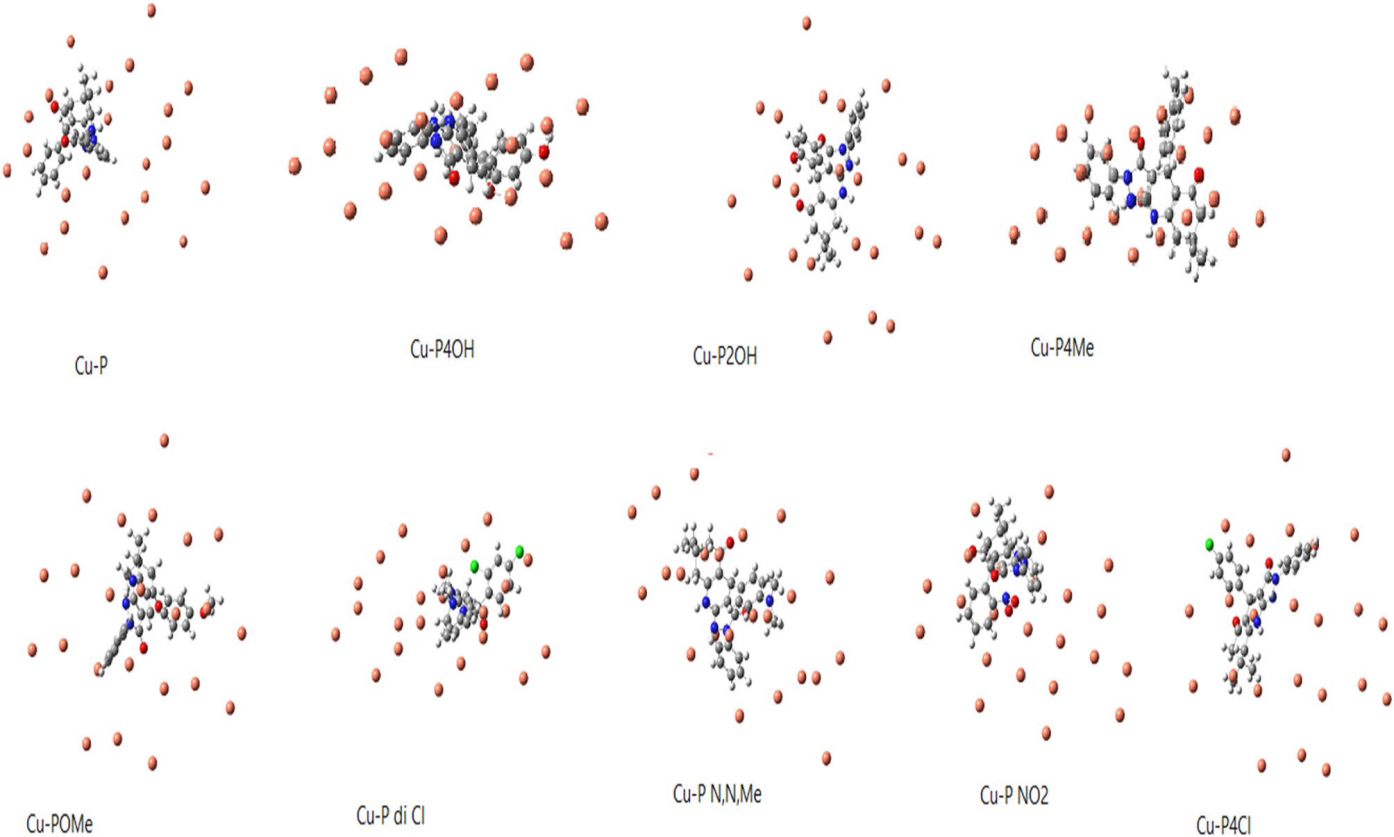
Optimized simulated Cu-Complexes of pyrazoloquinolines derivatives (https://chemistry.com.pk/software/free-download-chemdraw-ultra-12/, https://gaussview.software.informer.com/5.0/.

**Figure 5:**
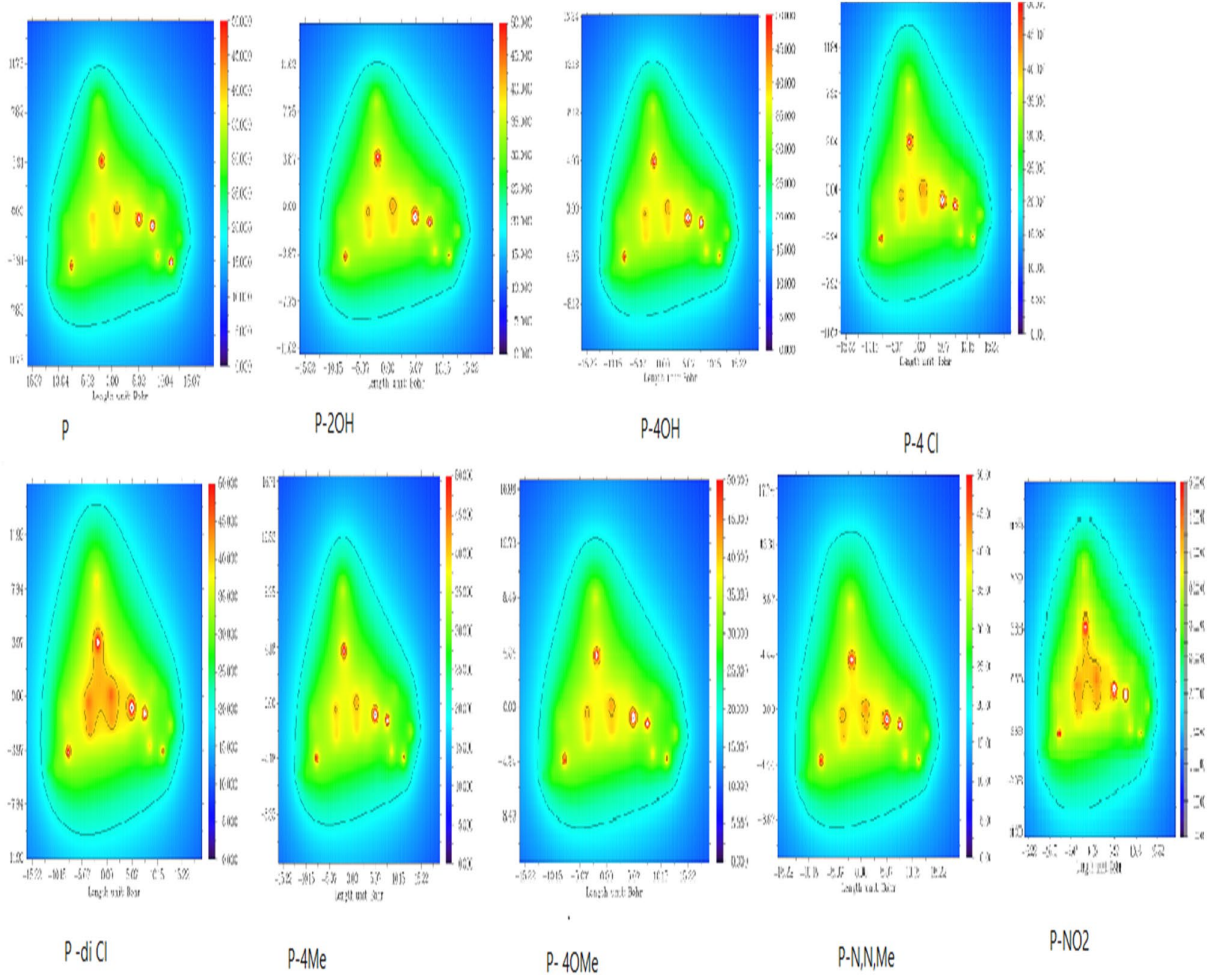
Electrostatic potential from nuclear charges of pyrazoloquinolines derivatives contours (https://www.wavefun.com/).

**Figure 6:**
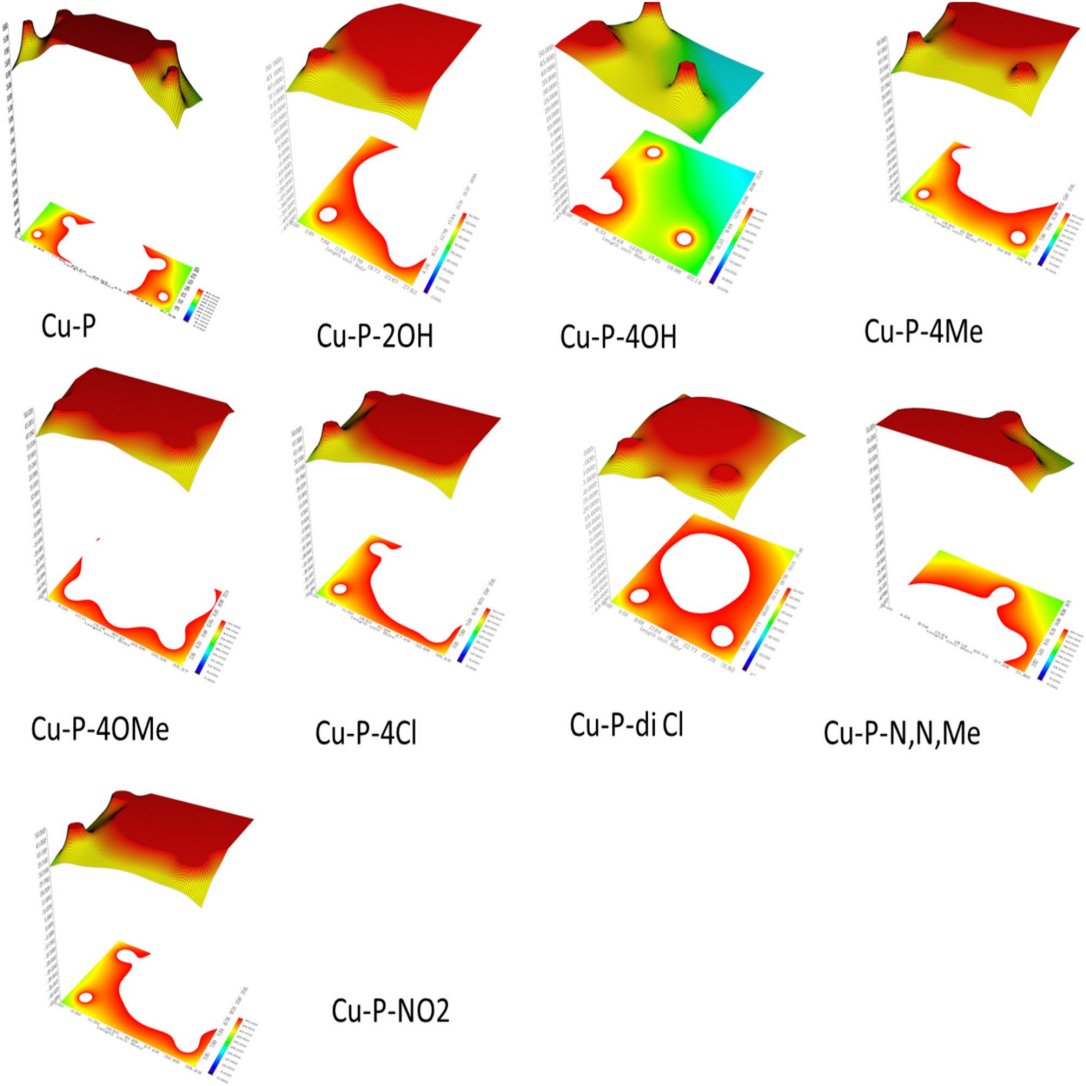
Electrostatic potential from nuclear charges of Cu complexes of pyrazoloquinolines derivatives profiles (https://www.wavefun.com/).

As indicated in Table 2, the molecules having –Ph, –OMe, –NO_2_, and –OH groups have high HOMO energy values. It can be seen from the equations presented above, chemical potential is the negative of the electronegativity. Electronegativity represents the electron withdrawal power of atoms, ions and molecules. It can be seen from the data presented in the related table that the derivatives containing –OH group have lower electronegativity values. These data imply that these molecules will be effective against the corrosion of metal surfaces. According to the results presented in Table 3, the molecules having –OMe and –OH functional groups have high dipole moments. It should be noted that dipole moment is often considered as a measure of polarizability. Therefore, the inhibitors including –OH and –OMe groups can act as effective inhibitors against the corrosion of copper surface^46^. Positive signs of numbers of dipole moment indicate that the inhibitors could be applied to the metal surface by physical mechanism^47^. All the pyrazoloquinoline derivatives have positive dipole moment so they have physical mechanism of inhibition. DFT effectively predicts the selectivity and reactivity in the light of the quantum molecular parameters such as chemical potential (*μ*) and electronegativity (*χ*)^48^. Another chemical parameter used is chemical hardness (*η*) defining as the resistance towards electron cloud polarization or deformation of molecules. According to HSAB Principle, “hard acids prefer to coordinate to hard bases and soft acids prefer to coordinate to soft bases” It is important to note that hard molecules have high HOMO–LUMO energy gap values. Softness is the multiplicative inverse of the chemical hardness. Soft molecules act effective electron donors^45^. Table 3 shows all the results related to the pyrazolo [3,4-b]quinoline-3,5-dione derivatives. It is seen that the molecules containing –OH, –OMe, and –NO_2_ have low values of chemical hardness. If so, one can say that they are more reactive compared to others. Figures 4 and 5 show the profile of charge density of pyrazoloquinoline derivatives and the profile of electrostatic potential Fig. 6 from nuclear charges in Cu complexes of pyrazoloquinoline derivatives, respectively. Increasing in the electron discharging power was exchanged by electron-donating molecules like (–OCH3 and –OH group) that occurred to improve prohibition but, electron-attracting group (–Cl) in pyrazoloquinoline derivatives decreases the effect of prohibition. There are two important quantum functions including electron-localization function and Laplacian density of electron exposing the electron donations linked with the amount of spatial structure arrangement of pairs in the localized electron implicitly in quantum model of VSEPR. Here, both experimental and theoretical electron densities was predicted and commented. Potential local energies and electronic kinetic were used for training the bonding in chemical compounds and lattices of metal crystals^49^. The power of bonding was determined by topological parameters There are numerous geometrical principles for determining the distance of bonds between Cu surface and chemical inhibitor molecules^30^. The QTAIM was established by electron density (ρ) related to attendance of (3, − 1) and (+ 3, − 3) BCPs for the proton inhibitors principally. Acceptor atom of Cu surface interaction validates bonding interaction and the range of electron density is from 0.002 to 0.04 a.u. Laplacian corresponding density must be equal to 0.024–0.139 a.u. Table 4 presents the results regarding calculating the topological factors of the desired bonds like ∇^2^ρ and ρ. Value of + 0.66 for –OH group in the pyrazolo [3,4-b]quinoline-3,5-dione derivatives showed active BCP for making reaction.

**Table 4:** Topological parameters of pyrazoloquinolines derivatives.

R-Complexes	BCP	*ρ*	${\nabla }^{2}\rho$
Ph	a	0.2644	− 0.79
	b	0.2635	− 0.783
	c	0.2639	− 0.787
2-(NO_2_)C_6_H_4_	a	0.2202	− 0.3293
	b	0.3194	− 0.7386 × 10^–1^
	c	0.3198	− 0.7501 × 10^–1^
4-(N,N-di-Me)C_6_H_4_	a	0.2422	− 0.5169
	b	0.2352	− 0.4886
	c	0.2719	− 0.8282
2,4-di-ClC_6_H_3_	a	0.2957	− 0.6874
	b	0.1849 × 10^–1^	− 0.7447 × 10^–1^
	c	0.1669	− 0.1449
2-(OH)C_6_H_4_	a	0.2340	− 0.85
	b	0.2369 × 10^–2^	+ 0.66
	c	0.3427	− 0.42
4-(OH)C_6_H_4_	a	0.4364	− 0.8739
	b	0.2911 × 10^+3^	− 0.4015
	c	0.4157	− 0.1654 × 10
4-(Me)C_6_H_4_	a	0.2689	− 0.8028
	b	0.2681	− 0.8028
	c	0.2688	− 0.7975
4-(OCH_3_)C_6_H_4_	a	0.1879	− 0.2055
	b	0.2267	− 0.2934
	c	0.2760	− 0.8688
4-(Cl)C_6_H_4_	a	0.2722	− 0.8442
	b	0.2748	− 0.8748
	c	0.3190	− 0.8185

Formation of the complexes of pyrazolo [3,4-b]quinoline-3,5-dione derivatives with Cu (fcc) surface atom was studied by the mentioned procedures. Due to energies of interaction and simulation sides of reaction, inhibitor molecules were placed in perpendicular orientation with the Cu surface. Calculations and analyses made shows that inhibitor molecules interact via the heteroatoms like O and N with metal surface.

QTAIM indicators were simulated as quantitative proofs to understand the environment of bond interactions between Cu surface and inhibitor molecules. Results of the QTAIM simulation showed the powerful interactions formed between Cu surface and inhibitors including –OH group Dipole moments depicted that polarity and solubility of the chemical complexes increase by adding chemical inhibitors to the Cu surface. Theoretical calculations supported that inhibitor molecules with –OH, –NO_2_ and –OMe group functions act as powerful corrosion inhibitors.

### Computational method

Optimized geometries were obtained via B3LYP/6‐311G++(d,p) calculation level of Gaussian 09 program^50^. Topological studies were performed to see the electron density of the surfaces. Stoichiometric ratio of 1:1 was considered for Cu complexes of pyrazolo [3,4-b]quinoline-3,5-dione derivatives. Virial theorem was applied to investigate the BCPs. The characteristics including density of electron (ρ) and (∇^2^ρ) were measured at BCP. Highest occupied molecular orbital and lowest unoccupied molecular orbital and all quantum chemical parameters related to the energy levels of frontier orbitals were calculated and discussed as detailed. Thermal energies were computed. Some thermodynamic parameters regarding to the interaction between copper and inhibitor molecules were analyzed. The polarizable continuum model (PCM) was employed for solvent effects.
